# Association between Sleep Disturbances at Subacute Stage of Mild Traumatic Brain Injury and Long-Term Outcomes

**DOI:** 10.1089/neur.2022.0004

**Published:** 2022-07-15

**Authors:** Shiyu Tang, Chandler Sours Rhodes, Li Jiang, Hegang Chen, Steven Roys, Neeraj Badjatia, Prashant Raghavan, Jiachen Zhuo, Rao P. Gullapalli

**Affiliations:** ^1^ Department of Diagnostic Radiology and Nuclear Medicine, Department of Neurology, University of Maryland School of Medicine, Baltimore, Maryland, USA.; ^2^ Center for Advanced Imaging Research (CAIR), Department of Neurology, University of Maryland School of Medicine, Baltimore, Maryland, USA.; ^3^ National Intrepid Center of Excellence, Walter Reed National Military Medical Center, Bethesda, Maryland, USA.; ^4^ Department of Epidemiology and Public Health, Department of Neurology, University of Maryland School of Medicine, Baltimore, Maryland, USA.; ^5^ Neurology Program and Trauma, Department of Neurology, University of Maryland School of Medicine, Baltimore, Maryland, USA.

**Keywords:** mild traumatic brain injury, neurocognitive functions, post-concussion symptoms, sleep disturbances

## Abstract

Mild (mTBI) traumatic brain injury (TBI) accounts for the majority of all TBI cases. Evidence has suggested that patients with mTBI can suffer from long-lasting cognitive deficits, persistent symptoms, and decreased quality of life. Sleep disorders are commonly observed after TBI, with the prevalence rate of sleep disturbances in persons with TBI being much higher than that in the general population. Poor sleep quality can impair cognitive functions in the general population. This effect of sleep disturbances may impede the recovery processes in the population with TBI. The objective of this study is to add to our understanding of the relationship between self-reported sleep problems and other post-concussion symptoms and look at the association between early sleep problems and long-term outcomes in mTBI. Post-concussion symptoms, neurocognitive functions, level of global outcomes, and rating of satisfaction of life were assessed in 64 patients with mTBI. The results revealed that the presence of sleep disturbances co-occur with an increased level of overall post-concussion symptoms at the subacute stage of mTBI, particularly with symptoms including poor concentration, memory problems, and irritability. In addition, sleep disturbance at the subacute stage is associated with persistent poor concentration and memory problems, as well as worse neurocognitive function, slower overall recovery, and lower satisfactory of life at the long term. Our findings suggest that sleep disturbance can be a prognostic factor of long-term outcomes after mTBI. Early interventions to improve sleep quality can have potential benefits to facilitate the recovery process from mTBI.

## Introduction

Traumatic brain injury (TBI) is the leading cause of the death and disabilities among all trauma-related injuries.^1^ Mild TBI (mTBI) accounts for ∼70–90% of all TBI cases.^2^ Despite being labeled as “mild” injuries, evidence has suggested that, in a subset of patients, mTBI may lead to long-lasting cognitive deficits, persistent symptoms, and decreased quality of life.^3–5^ Sleep disorders are commonly reported after TBI, with reports of 30–70% of TBI patients experiencing short- or long-term sleep problems post-injury.^6–8^ The prevalence rate of sleep disturbances in persons post-TBI is much higher than that in the general population^6^; however, the severity of sleep disturbances is not associated with severity of TBI.^9^ Patients with TBI across the entire range of severities are at risk. Sleep problems with insomnia and waking up too early were more frequently reported by mTBI patients than moderate or severe TBI patients.^10–12^ On the other hand, hypersomnia was found to be more profound in moderate-severe TBI patients.^13,14^

Previous studies showed that mTBI patients experiencing sleep problems often suffered from more severe overall post-concussion symptoms no matter the post-injury stage (i.e., acute, subacute, or chronic).^15,16^ However, the relationship between sleep and other individual post-concussion symptoms has not been widely investigated. Poor sleep quality is known to impair cognitive functions in the general population.^17,18^ These detrimental effects can delay the recovery process and exacerbate unfavorable outcomes post-TBI. For instance, mTBI patients experiencing poor sleep quality had a longer recovery period,^19^ and mTBI patients with persistent sleep problems at 1 year post-injury had significantly poorer long-term functional and social outcomes compared to those whose acute sleep problems recovered by 1 year post-injury.^20^

A study on neuropsychiatric outcomes reported that sleep disturbance presenting within 3 months post-injury was associated with increased depression, apathy, and anxiety 1 year after injury.^21^ Another study revealed a bidirectional relationship between sleep disturbances and poor global function across the first 6 months post-injury in a group of patients where the majority were mTBI patients.^22^ Sleep difficulties within 2 weeks of injury were shown to be highly associated with more severe post-concussion symptoms, poorer neuropsychiatric outcomes, and reduced overall neurocognitive functions 1 year after injury.^23^

The objective of this study is to examine sleep symptoms of mTBI patients and further our understanding of the relationship between sleep difficulties and post-concussion symptoms, as well as investigate the association between subacute sleep difficulties and long-term outcomes. We hypothesize that early sleep disruptions are associated with unfavorable long-term outcomes post-mTBI, including overall level of post-concussion symptoms, global neurocognitive function, recovery status, and satisfaction with life. In addition, we evaluated the association of early sleep disruptions and secondary measures, including individual post-concussion symptoms and neurocognitive functions.

## Methods

### Participants

mTBI patients were recruited from the R. Adam Cowley Shock Trauma Center at the University of Maryland Medical Center as part of the MagNeTs study (Magnetic Resonance Imaging of NeuroTraumaStudy), which included longitudinal behavioral and imaging assessment that spanned over 18 months.^24^ mTBI was defined as having an admission Glasgow Coma Scale of 13–15 and 1) a positive clinical computed tomography or 2) a mechanism of injury consistent with trauma and reporting loss of consciousness, alteration of consciousness, or post-traumatic amnesia. Exclusion criteria were: 1) history of neurological disorders; 2) pre-existing psychological disorders not including depression and anxiety disorders; 3) seizure disorders; 4) history of previous brain injury requiring hospitalization; and 5) contraindications to magnetic resonance imaging (MRI) since this study. Sixty-four mTBI patients were included in this study. A summary of patient information is shown in Table 1. All study procedures were approved by the institutional review board at the University of Maryland, and all participants provided written informed consent and Health Insurance Portability and Accountability Act (HIPAA) compliance.

**Table 1.** Summary of Patients with and without Sleep Disruption at Subacute Stage Post-mTBI

	All	Sleep disruption	
		Yes	No
*N* (male)	64 (47)	15 (9)	49 (38)
Age, years	43.8 ± 2.1	43.1 ± 3.5	43.9 ± 2.5
Education, years (mean ± SE)	13.8 ± 0.3	12.9 ± 0.7	14.0 ± 0.4
Long-term total RPQ (mean ± SE)	15.0 ± 2.1	31.9 ± 5.0	9.2 ± 1.8
Long-term ANAM WT (mean ± SE)	199.5 ± 5.5	174.0 ± 11.8	207.3 ± 5.8
Long-term GOSE score (mean ± SE)	7.3 ± 0.1	6.6 ± 0.3	7.5 ± 0.1
Long-term GOSE recovered (GOSE, >6)	49 (76.6%)	7 (46.7%)	42 (87.5%)
Long-term SWLS score (mean ± SE)	23.6 ± 1.0	18.9 ± 2.3	20.1 ± 1.0

mTBI, mild traumatic brain injury; SE, standard error; RPQ, Rivermead Post Concussion Symptoms Questionnaire; ANAM WT, Automated Neuropsychological Assessment Metrics weighted throughput score; GOSE, Extended Glasgow Outcome Scale; SWLS, Satisfaction with Life Scale.

### Measures

#### Modified Rivermead Post Concussion Symptoms Questionnaire

Severity of sleep problems and other post-concussion symptoms were measured using the modified Rivermead Post Concussion Symptoms Questionnaire (RPQ) at the subacute stage (mean ± standard error [SE], 37.0 ± 1.3 days) and the chronic stage (6–18 months; mean ± SE, 220.0 ± 12.2 days) post-injury.^25^ Each symptom is rated on a scale from 0 to 4 (0 = not experienced at all; 1 = no more of a problem; 2 = a mild problem; 3 = a moderate problem; and 4 = a severe problem). The modified RPQ used in the present study was adapted from the original 16-item RPQ by adding six additional items that were occasionally reported as “other difficulties” by mTBI patients (i.e., vertigo, loss of balance, hearing difficulty, numbness or tingling, changes in taste and/or smell, and difficulty making decisions). In the present study, the presence of sleep problems or any other post-concussion symptoms was defined as a rating >2 (moderate-severe) on the symptoms.

#### Automated Neuropsychological Assessment Metrics

Neurocognitive functions were assessed using the Automated Neuropsychological Assessment Metrics (ANAM) at the chronic stage of mTBI.^26,27^ The ANAM consists of seven subtests, that is, simple reaction time (SR), code substitution–learning (CS), procedural reaction time (PRT), mathematical processing (MATH), matching to sample (MTS), code substitution–delayed (CSD), and repeated simple reaction time (SR2).^26^ Accuracy and reaction time in each subtest were measured by a throughput score, which is the number of correct responses per minute. Overall performance was assessed using a weighted throughput score.^24^

#### Extended Glasgow Outcome Scale

The Extended Glasgow Outcome Scale (GOSE) was administered at the chronic stage of mTBI, which is used for measuring the level of disability and global outcomes post-TBI.^28^ According to the response in GOSE, subjects were assigned to one of eight categories: dead, vegetative state, lower severe disability, upper severe disability, lower moderate disability, upper moderate disability, lower good recovery, and upper good recovery. A dichotomization threshold was applied to divide patients into two groups: favorable (GOSE, >6; lower good recovery and upper good recovery) and unfavorable (GOSE, < = 6; the other six categories).

#### Satisfaction with Life Scale

Life satisfaction was measured with the Satisfaction with Life Scale (SWLS), which is a five-item, seven-point scale questionnaire at the chronic stage of mTBI.^29^ On each item, participants rate their agreement from strongly disagree (point 1) to strongly agree (point 7). Higher total score is indicative of higher satisfaction with life.

#### Pittsburgh Sleep Quality Index

Detailed sleep quality was assessed in a subset of patients (*n* = 30) using the Pittsburgh Sleep Quality Index (PSQI).^30^ A summary of patient information is shown in Table 2. The PSQI contains 19 self-rated items that measure seven components of sleep quality (i.e., subjective sleep quality, sleep latency, sleep duration, habitual sleep efficiency, sleep disturbances, use of sleep medications, and daytime dysfunction). A global score >8 was applied to distinguish patients having good versus poor sleep as proposed by Fichtenberg and colleagues.^31^

**Table 2.** Summary of Patients in the Subset with PSQI Assessment

		All	Sleep disruption	
			Yes	No
*N* (male)		30 (23)	9 (3)	21 (20)^^***^^
Age, years		47.2 ± 2.9	42.1 ± 5.8	49.4 ± 3.3
Education, years (mean ± SE)		14.0 ± 0.5	13.1 ± 0.8	14.4 ± 0.7
Long-term total RPQ (mean ± SE)		16.9 ± 3.1	27.3 ± 6.7	12.4 ± 3.1
Long-term ANAM WT (mean ± SE)		194.6 ± 7.3	187.6 ± 14.3	197.6 ± 8.6
Long-term GOSE score (mean ± SE)		7.0 ± 0.2	6.2 ± 0.4	7.4 ± 0.2
Long-term GOSE recovered (GOSE, >6)		22 (73.3%)	4 (44.4%)	18 (85.7%)
Long-term SWLS score (mean ± SE)		23.3 ± 1.4	19.3 ± 2.6	25.1 ± 1.6

^***^ *p* < 0.001, Fisher's exact test comparing patients with and without sleep disruption during the subacute injury stage.

PSQI, Pittsburgh Sleep Quality Index; SE, standard error; RPQ, Rivermead Post Concussion Symptoms Questionnaire; ANAM WT, Automated Neuropsychological Assessment Metrics weighted throughput score; GOSE, Extended Glasgow Outcome Scale; SWLS, Satisfaction with Life Scale.

### Statistical analysis

Data analysis was performed using R Statistical Software (version 4.0.2; The R Foundation for Statistical Computing, Vienna, Austria). All patients completed the RPQ, ANAM, GOSE assessment, and SWLS. Years of education of 3 patients were missing. These 3 patients were not included in the regression analysis described below.

#### Demographic information

Demographic information was compared between patients with and without sleep problem at subacute stage of injury. Fisher's exact test was used to compare the sex composition between the two groups. A two-sample *t*-test was performed to compare age and years of education.

#### Sleep problems and post-concussion symptoms

RPQ total score without the sleep scale was used to reflect the overall level of post-concussion symptoms. Linear regressions were performed to assess the relationship between sleep problems at the subacute stage and overall post-concussion symptoms at the subacute and chronic stages. Headaches, dizziness, trouble concentrating, memory problems, fatigue, and irritability were used to define post-concussion syndrome together with sleep problems.^32^ Sleep difficulties often co-occur with post-concussion symptoms.^15,16^ Logistic regression was performed to assess the relationship between the presence of sleep problems and the additional six post-concussion symptoms at the subacute stage of injury. Another logistic regression analysis was performed to investigate whether early sleep problems impose an extra risk of persistent post-concussion symptoms at the chronic stage after controlling for the corresponding symptoms at the subacute stage. Age, sex, and years of education were added to the analysis as independent variables.

#### Early sleep problem and long-term outcomes

Linear regressions were performed using the presence of sleep problem at the subacute stage, age, years of education, and sex as independent variables and outcome measures at the chronic stage of injury as dependent variables (i.e., ANAM weighted throughput score, GOSE score, and SWLS total score). The association between sleep problems and recovery status (favorable vs. unfavorable as assessed by the GOSE) was assessed using a logistic regression, with the dichotomized GOSE as the dependent variable and the same set of independent variables as in the linear regression. In addition, a profile analysis across the seven ANAM subtests was performed to assess whether patients with and without subacute sleep problems would demonstrate different performance patterns in the tests. Linear regressions were then performed on the throughput score of each subtest to identify subtests that are highly associated with early sleep problems.

#### Sleep-quality evaluation with the Rivermead Post Concussion Symptoms Questionnaire and Pittsburgh Sleep Quality Index

To evaluate the consistency of the RPQ and PSQI in detecting the presence of sleep problems at the subacute stage, a Fisher's exact test was performed to compare the number of patients with and without sleep problems as detected with the RPQ and PSQI. To characterize the association between the RPQ sleep scale and each component in the PSQI, cumulative link models were performed on the score of each of the seven components, with the RPQ sleep score and sex as independent variables. Years of education was not significant for any of the components and was not expected to affect the association between the RPQ and PSQI and therefore was excluded from the model.

## Results

### Demographic information

Sex composition (*p* = 0.197), age (*p* = 0.848), and years of education (*p* = 0.188) were not significantly different between patients with and without sleep problem at the subacute stage (Table 1). In the subset of patient with the PSQI assessment, significantly more females presented sleep problems compared to males (*p* < 0.001; Table 2). Age (*p* = 0.252) and years of education (*p* = 0.268) were not significantly different between patients with and without sleep problem in this subset (Table 2).

### Relationship between sleep problems and post-concussion symptoms

Patients with moderate-severe sleep problems at the subacute stage suffered from more severe post-concussion symptoms during the subacute stage of injury according to the RPQ total score (*p* < 0.001; Table 3). They also had a significantly higher odds of concurrently experiencing trouble concentrating (*p* = 0.020), memory problems (*p* = 0.015), and irritability (*p* = 0.009) and a trend for increased risk of headaches (*p* = 0.071) and fatigue (*p* = 0.05; Table 3). Similarly, patients who reported early sleep problems were more likely to experience more severe chronic post-concussion symptoms (*p* = 0.032) regardless of the level of overall post-concussion symptoms at the subacute stage (Table 4). The presence of early sleep problems at the subacute stage significantly increased the chance of experiencing trouble concentrating (*p* = 0.002) and memory problems (*p* = 0.038) at the chronic stage whether or not these symptoms were presented at the subacute stage (Table 4).

**Table 3.** Relationship between Sleep Disruption and Post-Concussion Symptoms at Subacute Stage Post-Injury

RPQ	Sleep disruption		Age		Sex		Years of education	
	Coefficient (95% CI)	*p* value	Coefficient (95% CI)	*p* value	Coefficient (95% CI)	*p* value	Coefficient (95% CI)	*p* value
RPQ total^a^	19.36 (11.83, 26.89)	<0.001	0.06 (−0.14, 0.26)	0.553	–6.21 (−13.34, 0.92)	0.086	–0.83 (−2.06, 0.40)	0.184
Headaches	1.43 (−0.13, 3.04)	0.071	–0.01 (−0.06, 0.04)	0.816	–0.26 (−1.90, 1.52)	0.755	–0.28 (−0.73, 0.06)	0.148
Dizziness	0.10 (−2.06, 1.94)	0.917	0.00 (−0.05, 0.05)	0.970	–1.15 (−3.01, 0.68)	0.203	–0.31 (−0.91, 0.09)	0.194
Trouble concentrating	1.62 (0.26, 3.03)	0.020	0.00 (−0.04, 0.04)	0.993	–0.93 (−2.33, 0.47)	0.185	–0.07 (−0.36, 0.20)	0.644
Memory problems	2.20 (0.52, 4.18)	0.015	–0.01 (−0.07, 0.04)	0.666	0.69 (−1.10, 2.93)	0.489	0.07 (−0.26, 0.41)	0.662
Fatigue	1.38 (−0.01, 2.80)	0.050	–0.01 (−0.05, 0.04)	0.725	–0.63 (−2.03, 0.82)	0.373	–0.03 (−0.32, 0.23)	0.825
Irritability	3.57 (1.33, 7.04)	0.009	–0.04 (−0.13, 0.03)	0.316	0.60 (−1.58, 3.27)	0.616	0.15 (−0.29, 0.65)	0.505

^a^ Measurements from linear regression. Other measurements were from logistic regression.

RPQ, Rivermead Post Concussion Symptoms Questionnaire; 95% CI, 95% confidence interval.

**Table 4.** Relationship between Subacute Sleep Disruption with Other Post-Concussion Symptoms and Outcome Measures at Chronic Stage

Assessments	Sleep disruption		Age		Sex		Years of education	
	Coefficient (95% CI)	*p* value	Coefficient (95% CI)	*p* value	Coefficient (95% CI)	*p* value	Coefficient (95% CI)	*p* value
RPQ								
RPQ total^a^	8.20 (0.72, 15.68)	0.032	0.15 (−0.01, 0.31)	0.073	1.28 (−4.71, 7.28)	0.670	–0.43 (−1.46, 0.59)	0.399
Headaches	2.09 (−0.49, 5.65)	0.144	–0.03 (−0.15, 0.06)	0.483	–1.06 (−4.55, 1.87)	0.473	0.07 (−0.58, 0.71)	0.827
Dizziness	1.61 (−0.16, 3.49)	0.074	0.02 (−0.04, 0.08)	0.538	0.27 (−1.53, 2.41)	0.780	–0.06 (−0.46, 0.30)	0.753
Trouble concentrating	3.16 (1.38, 5.50)	0.002	–0.02 (−0.10, 0.04)	0.472	0.80 (−1.22, 3.15)	0.463	–0.10 (−0.53, 0.27)	0.600
Memory problems	1.80 (0.13, 3.62)	0.038	0.05 (0.00, 0.12)	0.070	0.83 (−0.88, 2.84)	0.369	–0.21 (−0.58, 0.07)	0.188
Fatigue	2.11 (−0.05, 4.77)	0.072	–0.03 (−0.12, 0.05)	0.461	0.04 (−2.31, 2.65)	0.976	0.04 (−0.50, 0.54)	0.858
Irritability	1.78 (−0.33, 4.08)	0.096	0.03 (−0.03, 0.11)	0.356	–0.81 (−2.98, 1.25)	0.432	–0.32 (−0.93, 0.10)	0.198
ANAM^a^								
WT	–29.21 (−48.67, −9.75)	<0.001	–1.62 (−2.13, −1.12)	0.004	1.12 (−17.30, 19.55)	0.903	4.34 (1.16, 7.53)	0.008
CS	–6.61 (−13.01, −0.20)	0.043	–0.54 (−0.71, −0.37)	<0.001	–4.02 (−10.08, 2.04)	0.189	0.86 (−0.19, 1.91)	0.106
CSD	–6.96 (−15.14, 1.22)	0.094	–0.681 (−0.89, −0.47)	<0.001	–3.15 (−10.89, 4.60)	0.420	0.775 (−0.56, 2.11)	0.251
MTS	–4.21 (−9.80, 1.39)	0.138	–0.24 (−0.39, −0.10)	0.001	–0.12 (−5.42, 5.18)	0.963	0.78 (−0.13, 1.70)	0.092
MATH	–2.82 (−7.02, 1.38)	0.183	–0.03 (−0.14, 0.08)	0.552	3.09 (−0.89, 7.07)	0.125	0.91 (0.23, 1.60)	0.01
PRT	–5.79 (−17.02, 5.44)	0.306	–0.35 (−0.64, −0.06)	0.02	1.93 (−8.70, 12.57)	0.717	1.64 (−0.20, 3.48)	0.079
SR	–37.66 (−61.68, −13.64)	0.003	–1.41 (−2.04, −0.79)	<0.001	7.17 (−15.58, 29.91)	0.530	1.86 (−2.07, 5.79)	0.35
SR2	–39.95 (−63.16, −16.75)	0.001	–1.62 (−2.22, −1.01)	<0.001	3.78 (−18.19, 25.76)	0.731	4.65 (0.86, 8.45)	0.017
GOSE								
Score^a^	–0.75 (−1.38, −0.13)	0.020	–0.01 (−0.03, 0.00)	0.101	0.47 (−0.13, 1.06)	0.121	0.06 (−0.04, 0.16)	0.263
Favorable recovery	–1.79 (−3.25, −0.44)	0.011	–0.03 (−0.08, 0.01)	0.132	1.07 (−0.31, 2.49)	0.127	0.16 (−0.11, 0.48)	0.267
SWLS total^a^	–5.59 (−10.35, −0.84)	0.005	–0.04 (−0.17, 0.08)	0.516	0.30 (−4.20, 4.80)	0.894	0.67 (−0.11, 1.44)	0.092

^a^ Measurements from linear regression. Other measurements were from logistic regression.

RPQ, Rivermead Post Concussion Symptoms Questionnaire; ANAM WT, Automated Neuropsychological Assessment Metrics weighted throughput score; CS, code substitution–learning; CSD, code substitution–delayed; MATH, mathematical processing; MTS, matching to sample; PRT, procedural reaction time; SR, simple reaction time; SR2, repeated simple reaction time; GOSE, Extended Glasgow Outcome Scale; SWLS, Satisfaction with Life Scale.

### Association between early sleep problems and long-term outcomes

**FIG. 1.**
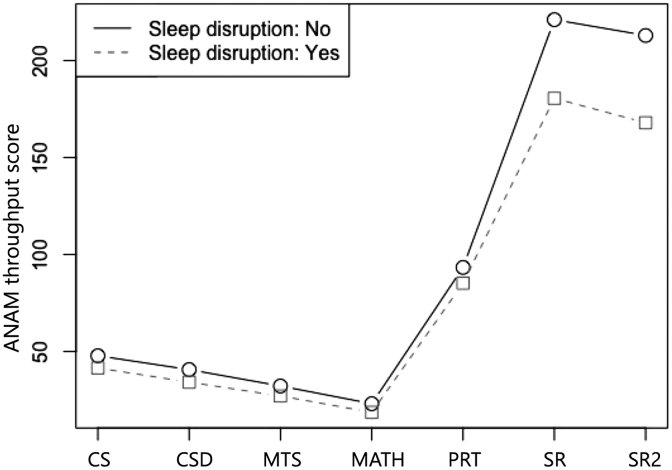
Profile plot showing throughput scores of the seven ANAM subtests for patients with and without early sleep problems. ANAM, Automated Neuropsychological Assessment Metrics; CS, code substitution–learning; CSD, code substitution–delayed; MATH, mathematical processing; MTS, matching to sample; PRT, procedural reaction time; SR, simple reaction time; SR2, repeated simple reaction time.

Global neurocognitive performance, as reflected by the ANAM weighted throughput at the chronic stage, was significantly lower in patients reporting early sleep problems (*p* < 0.001; Table 4). Overall performance on the ANAM battery was also affected by age and years of education, with younger, more highly educated patients performing better. The profile analysis showed a trend of interaction between the patient group and subtests (test of parallelism, *p* = 0.086), indicating possible differences in the performance pattern between patients with and without early sleep problems. Test of equality suggested significant differences in subtests between the two groups of patients (*p* = 0.003; Fig. 1). Specifically, significant negative effects of early sleep problems were observed on the CS (*p* = 0.043), SR (*p* = 0.003), and SR2 (*p* = 0.001) subtests, indicating lower functions in attention, visual perception, visuomotor response, and mental fatigue (Table 4). Significant negative effects of age were observed on all but the MATH subtests, where older patients had worse neurocognitive performance. Years of education showed positive effects on MATH and SR2, where patients with higher education performed better in MATH and SR2 (Table 4).

Subacute moderate-severe sleep problems were significantly associated with lower GOSE score (*p* = 0.02) and lower SWLS (*p* = 0.005) at the chronic stage. Logistic regression on recovery status showed that patients with subacute sleep problems were less likely to have favorable recovery at the chronic stage (*p* = 0.011; Table 4).

### Consistency between Rivermead Post Concussion Symptoms Questionnaire Sleep Scale and Pittsburgh Sleep Quality Index

Results showed a consistency between the presence of moderate-severe sleep problems reported on the RPQ sleep scale and PSQI (*p* = 0.003; Table 5). Among the seven components of the PSQI, the presence of sleep problem as detected by the RPQ was significantly associated with issues in sleep duration (*p* = 0.016), sleep disturbances (*p* = 0.029), sleep latency (*p* = 0.006), habitual sleep efficiency (*p* = 0.031), and subjective sleep quality (*p* = 0.003; Table 6). The PSQI components that showed no association with RPQ-detected sleep problems were daytime dysfunction (*p* = 0.079) and sleep medications (*p* = 0.378; Table 6).

**Table 5.** Co-occurrence of Sleep Problem as Detected with RPQ and PSQI at Subacute Stage Post-Injury

RPQ vs. PSQI		Sleep disruption (PSQI)	
		No	Yes
Sleep disruption (RPQ)	No	19	3
	Yes	2	6

RPQ, Rivermead Post Concussion Symptoms Questionnaire; PSQI, Pittsburgh Sleep Quality Index.

**Table 6.** Association between Sleep Disruption as Detected with RPQ and PSQI Components at Subacute Stage Post-Injury

PSQI components	Sleep disruption (RPQ)		Age		Sex	
	Coefficient (95% CI)	*p* value	Coefficient (95% CI)	*p* value	Coefficient (95% CI)	*p* value
Sleep duration	2.19 (0.49, 4.12)	0.016	–0.01 (−0.06, 0.04)	0.729	–1.13 (−3.02, 0.64)	0.214
Sleep disturbances	2.67 (0.50, 5.46)	0.029	0.07 (0.01, 0.15)	0.038	0.82 (−1.44, 3.30)	0.484
Sleep latency	2.61 (0.83, 4.64)	0.006	–0.02 (−0.07, 0.02)	0.337	–0.69 (−2.64, 1.16)	0.465
Daytime dysfunctions	1.80 (−0.09, 4.05)	0.079	0.00 (−0.04, 0.05)	0.844	–1.87 (−4.15, 0.04)	0.071
Habitual sleep efficiency	1.82 (0.20, 3.57)	0.031	–0.01 (−0.05, 0.04)	0.734	–1.13 (−2.93, 0.58)	0.195
Subjective sleep quality	4.06 (1.74, 7.44)	0.003	–0.03 (−0.08, 0.02)	0.253	–3.35 (−6.59, −1.08)	0.011
Sleep medications	0.91 (−1.21, 2.97)	0.378	0.05 (−0.01, 0.13)	0.120	–2.30 (−4.86, −0.11)	0.049

RPQ, Rivermead Post Concussion Symptoms Questionnaire; PSQI, Pittsburgh Sleep Quality Index; 95% CI, 95% confidence interval.

## Discussion

Sleep loss or prolonged wakefulness can affect cognitive capacities and impair quality of life. Previous studies reported attention deficits, memory problems, and impaired executive functions after either total or partial sleep deprivation.^17,33,34^ It is known that patients with mTBI can suffer from long-term cognitive difficulties and lower quality of life. This study showed that moderate-severe sleep disturbances are associated with delayed recovery of cognitive functions and unfavorable long-term outcomes post-mTBI.

In the present study, presence of sleep disturbances was accompanied by an increased level of overall post-concussion symptoms at the subacute stage of mTBI. In particular, sleep disturbances tend to occur simultaneously with poor concentration, memory problems, and irritability. In addition, the presence of subacute sleep disturbances is also associated with persistent poor concentration and memory problems, as well as reduced neurocognitive performance, global recovery, and satisfaction with life in the chronic stage.

The role of sleep in memory has been widely investigated in the general population. Current theory suggests that sleep actively enhances the consolidation of memory in addition to passively protecting memory from interfering stimuli.^35^ Different stages of sleep have unique contributions to memory consolidation. For example, slow-wave sleep (SWS) can enhance declarative memory consolidation whereas rapid eye movement (REM) sleep can improve implicit memory processes.^36–38^ Other studies have suggested that SWS and REM play complementary roles in neural reactivation and synaptic remodeling for the transition from short- to long-term storage of memory.^35,39^ Reduced REM and increased SWS percentages have been reported in patients at the subacute to chronic stages of TBI compared to the population without a history of TBI.^40–42^ This impaired balance between REM and SWS may disrupt sleep-dependent memory consolidation, which could contribute to long-term memory problems.

In addition to memory problems, the present study shows that sleep difficulty at the subacute stage may predict long-term difficulties in cognitive functions, such as attention, visual perception, and visuomotor function. This can be related with disrupted sleep-dependent synaptic regulation and plasticity.^43,44^ Sleep-dependent plasticity plays an important role in cognitive recovery after brain injuries. Sleep deprivation can lead to reduced axonal sprouting and neurogenesis in rats after focal cerebral ischemia.^45^ In contrast, enhancing slow waves during sleep can increase local axonal sprouting and cortical circuit reorganization in mice during stroke recovery.^46^ Good sleep quality can facilitate neural plasticity in response to brain injuries; however, having poor sleep quality is likely to impede this process and delay the recovery of cognitive functions such as attention and memory.

Besides neural plasticity, poor sleep quality may contribute to poor outcomes through disrupting the function of the glymphatic system. The sleep-dependent glymphatic system, most active during non-rapid-eye movement and SWS sleep,^47,48^ is a glial-dependent perivascular network.^49^ It communicates with meningeal lymphatic channels to drain brain waste through an exchange between cerebrospinal fluid and interstitial fluid.^50^ Impaired glymphatic clearance can lead to the accumulation of waste metabolites, such as tau protein and amyloid-beta, and contribute to the development of cognitive impairments.^49,51^ TBI is known to directly affect the glymphatic and meningeal lymphatic systems through vascular disruption and aquaporin-4 channel alteration.^52–54^

Recent studies suggested that sleep can play a moderator role on the effect of TBI.^55^ Sleep deprivation or restriction can result in the accumulation of amyloid-beta in both humans and rodents, indicating possible dysfunction of the glymphatic system.^56,57^ A study on veterans showed that mTBIs are more likely to result in glymphatic dysfunction in subjects with poor sleep, as evidenced by an increase in the number and volume of MRI-visible perivascular spaces, a part of the glymphatic pathway, compared to those with normal sleep.^58^ Disrupted glymphatic function could therefore be another factor connecting sleep difficulties and long-term outcomes observed in the present study.

Several limitations should be noted in this study. The sleep problems in this study were identified using the single self-reported item on the RPQ. Therefore, the sleep problems in this study can only be taken as a general description of subjective sleep quality, which is not as comprehensive as the PSQI. However, this study demonstrated an association between self-reported sleep problems reflected in the sleep scale of the RPQ and those detected with the PSQI in the subset of patients for which the PSQI was collected. Although this association was only measured in a small subset of participants, which may not be ideal for representing the overall mTBI population, it suggests that the RPQ can be a potential quick assessment of subjective sleep quality when a comprehensive evaluation is not feasible. Another limitation is that this study included only subjective measures of sleep. Future studies using objective measures (i.e., overnight sleep studies using polysomnography) should be performed to further investigate what aspects of sleep are disrupted. Further, the present study only includes patients with mTBI, and therefore the relationships between sleep disturbances and chronic outcomes were not investigated in subjects who had been injured but without TBI (i.e., orthopedic controls). It is not clear whether the pattern of relationships is common in the general population or is moderated by mTBI.

Future studies on objective sleep quality and physiological processes, such as neural plasticity and glymphatic function, in both mTBI and the general population are needed. In addition, our study is based on a limited sample size and therefore needs to be interpreted with caution. To evaluate the magnitude of the effect of subacute-stage sleep disruptions on our primary measures (i.e., total RPQ, ANAM weighted throughput, GOSE, and SWLS), we calculated the effect size using Cohen's *f*^2^. Cohen's *f*^2^ for total RPQ at the subacute and chronic stages were 0.47 and 0.09, respectively, indicating large- and small-to-medium effects. Cohen's *f*^2^ also indicated medium effects on ANAM weighted throughput (*f*^2^ = 0.16) and GOSE recovery status (*f*^2^ = 0.13) and small-to-medium effects on the SWLS (*f*^2^ = 0.1). The effect sizes suggest that the impact of subacute-stage sleep disruptions detected in this study is meaningful. However, future studies with larger sample sizes are needed to verify our findings in large populations.

## Conclusion

This study revealed relationships between early sleep problems and both short- and long-term outcomes in patients with mTBI. The results agree with previous reports that sleep disturbances are linked with impeded post-injury recovery. In addition, this study showed that the single scale of sleep quality from the RPQ could be a useful tool for the quick assessment of overall sleep quality and that it can be predictive to long-term neurocognitive function and overall recovery. Improvement of sleep quality early post-injury may facilitate recovery and increase the efficacy of other interventions. Future studies on the mechanisms underlying this sleep-injury interaction will provide therapeutic targets and contribute to treatment optimization.

## Abbreviations Used

ANAM: Automated Neuropsychological Assessment Metrics
CS: code substitution – learning
CSD: code substitution–delayed
GOSE: Extended Glasgow Outcome Scale
MATH: mathematical processing
MRI: magnetic resonance imaging
mTBI: mild traumatic brain injury
MTS: matching to sample
PRT: procedural reaction time
PSQI: Pittsburgh Sleep Quality Index
REM: rapid eye movement
RPQ: Rivermead Post Concussion Symptoms Questionnaire
SE: standard error
SR: simple reaction time
SR2: repeated simple reaction time
SWLS: Satisfaction with Life Scale
SWS: slow-wave sleep
TBI: traumatic brain injury

## References

[1] Rubiano, A.M., Carney, N., Chesnut, R., and Puyana, J.C. (2015). Global neurotrauma research challenges and opportunities. Nature 527, S193–S197. 26580327 10.1038/nature16035

[2] Cassidy, J.D., Carroll, L.J., Peloso, P.M., Borg, J., von Holst, H., Holm, L., Kraus, J., and Coronado, V.G.; WHO Collaborating Centre Task Force on Mild Traumatic Brain Injury. (2004). Incidence, risk factors and prevention of mild traumatic brain injury: results of the WHO Collaborating Centre Task Force on Mild Traumatic Brain Injury. J. Rehabil. Med. 43 Suppl., 28–60. 10.1080/16501960410023732 15083870

[3] Daneshvar, D.H., Riley, D.O., Nowinski, C.J., McKee, A.C., Stern, R.A., and Cantu, R.C. (2011). Long-term consequences: effects on normal development profile after concussion. Phys. Med. Rehabil. Clin. N. Am. 22, 683–700, ix. 22050943 10.1016/j.pmr.2011.08.009 PMC3208826

[4] McInnes, K., Friesen, C.L., MacKenzie, D.E., Westwood, D.A., and Boe, S.G. (2017). Mild Traumatic Brain Injury (mTBI) and chronic cognitive impairment: a scoping review. PLoS One 12, e0174847. 28399158 10.1371/journal.pone.0174847 PMC5388340

[5] Theadom, A., Parag, V., Dowell, T., McPherson, K., Starkey, N., Barker-Collo, S., Jones, K., Ameratunga, S., and Feigin, V.L.; BIONIC Research Group. (2016). Persistent problems 1 year after mild traumatic brain injury: a longitudinal population study in New Zealand. Br. J. Gen. Pract. 66, e16–e23. 26719482 10.3399/bjgp16X683161 PMC4684031

[6] Mathias, J.L., and Alvaro, P.K. (2012). Prevalence of sleep disturbances, disorders, and problems following traumatic brain injury: a meta-analysis. Sleep Med. 13, 898–905. 22705246 10.1016/j.sleep.2012.04.006

[7] Castriotta, R.J., Wilde, M.C., Lai, J.M., Atanasov, S., Masel, B.E., and Kuna, S.T. (2007). Prevalence and consequences of sleep disorders in traumatic brain injury. J. Clin. Sleep Med. 3, 349–356. 17694722 PMC1978308

[8] Kempf, J., Werth, E., Kaiser, P.R., Bassetti, C.L., and Baumann, C.R. (2010). Sleep-wake disturbances 3 years after traumatic brain injury. J. Neurol. Neurosurg. Psychiatry 81, 1402–1405. 20884672 10.1136/jnnp.2009.201913

[9] Baumann, C.R., Werth, E., Stocker, R., Ludwig, S., and Bassetti, C.L. (2007). Sleep-wake disturbances 6 months after traumatic brain injury: a prospective study. Brain 130, 1873–1883. 17584779 10.1093/brain/awm109

[10] Beetar, J.T., Guilmette, T.J., and Sparadeo, F.R. (1996). Sleep and pain complaints in symptomatic traumatic brain injury and neurologic populations. Arch. Phys. Med. Rehabil. 77, 1298–1302. 8976315 10.1016/s0003-9993(96)90196-3

[11] Clinchot, D.M., Bogner, J., Mysiw, W.J., Fugate, L., and Corrigan, J. (1998). Defining sleep disturbance after brain injury. Am. J. Phys. Med. Rehabil. 77, 291–295. 9715917 10.1097/00002060-199807000-00006

[12] Ouellet, M.C., Beaulieu-Bonneau, S., and Morin, C.M. (2006). Insomnia in patients with traumatic brain injury: frequency, characteristics, and risk factors. J. Head Trauma Rehabil. 21, 199–212. 16717498 10.1097/00001199-200605000-00001

[13] Hou, L., Han, X., Sheng, P., Tong, W., Li, Z., Xu, D., Yu, M., Huang, L., Zhao, Z., Lu, Y., and Dong, Y. (2013). Risk factors associated with sleep disturbance following traumatic brain injury: clinical findings and questionnaire based study. PLoS One 8, e76087. 24098425 10.1371/journal.pone.0076087 PMC3788026

[14] Watson, N.F., Dikmen, S., Machamer, J., Doherty, M., and Temkin, N. (2007). Hypersomnia following traumatic brain injury. J. Clin. Sleep Med. 3, 363–368. 17694724 PMC1978314

[15] Towns, S.J., Silva, M.A., and Belanger, H.G. (2015). Subjective sleep quality and postconcussion symptoms following mild traumatic brain injury. Brain Inj. 29, 1337–1341. 26288022 10.3109/02699052.2015.1045030

[16] Sullivan, K.A., Berndt, S.L., Edmed, S.L., Smith, S.S., and Allan, A.C. (2016). Poor sleep predicts subacute postconcussion symptoms following mild traumatic brain injury. Appl. Neuropsychol. Adult 23, 426–435. 27183274 10.1080/23279095.2016.1172229

[17] Alhola, P., and Polo-Kantola, P. (2007). Sleep deprivation: impact on cognitive performance. Neuropsychiatr. Dis. Treat. 3, 553–567. 19300585 PMC2656292

[18] Zavecz, Z., Nagy, T., Galko, A., Nemeth, D., and Janacsek, K. (2020). The relationship between subjective sleep quality and cognitive performance in healthy young adults: evidence from three empirical studies. Sci. Rep. 10, 4855. 32184462 10.1038/s41598-020-61627-6 PMC7078271

[19] Hoffman, N.L., O'Connor, P.J., Schmidt, M.D., Lynall, R.C., and Schmidt, J.D. (2020). Relationships between post-concussion sleep and symptom recovery: a preliminary study. J. Neurotrauma 37, 1029–1036. 31774024 10.1089/neu.2019.6761

[20] Chan, L.G., and Feinstein, A. (2015). Persistent sleep disturbances independently predict poorer functional and social outcomes 1 year after mild traumatic brain injury. J. Head Trauma Rehabil. 30, E67–E75. 25931180 10.1097/HTR.0000000000000119

[21] Rao, V., McCann, U., Han, D., Bergey, A., and Smith, M.T. (2014). Does acute TBI-related sleep disturbance predict subsequent neuropsychiatric disturbances? Brain Inj. 28, 20–26. 24328797 10.3109/02699052.2013.847210

[22] Kalmbach, D.A., Conroy, D.A., Falk, H., Rao, V., Roy, D., Peters, M.E., Van Meter, T.E., and Korley, F.K. (2018). Poor sleep is linked to impeded recovery from traumatic brain injury. Sleep 41, zsy147. 10.1093/sleep/zsy147 PMC6890523 30053263

[23] Theadom, A., Cropley, M., Parmar, P., Barker-Collo, S., Starkey, N., Jones, K., and Feigin, V.L.; BIONIC Research Group. (2015). Sleep difficulties one year following mild traumatic brain injury in a population-based study. Sleep Med. 16, 926–932. 26138280 10.1016/j.sleep.2015.04.013

[24] Sours, C., Zhuo, J., Roys, S., Shanmuganathan, K., and Gullapalli, R.P. (2015). Disruptions in resting state functional connectivity and cerebral blood flow in mild traumatic brain injury patients. PLoS One 10, e0134019. 26241476 10.1371/journal.pone.0134019 PMC4524606

[25] King, N.S., Crawford, S., Wenden, F.J., Moss, N.E., and Wade, D.T. (1995). The Rivermead Post Concussion Symptoms Questionnaire: a measure of symptoms commonly experienced after head injury and its reliability. J. Neurol. 242, 587–592. 8551320 10.1007/BF00868811

[26] Reeves, D.L., Bleiberg, J., Roebuck-Spencer, T., Cernich, A.N., Schwab, K., Ivins, B., Salazar, A.M., Harvey, S.C., Brown, F.H. Jr., and Warden, D. (2006). Reference values for performance on the Automated Neuropsychological Assessment Metrics V3.0 in an active duty military sample. Mil. Med. 171, 982–994. 17076451 10.7205/milmed.171.10.982

[27] Vincent, A.S., Roebuck-Spencer, T.M., Cox-Fuenzalida, L.E., Block, C., Scott, J.G., and Kane, R. (2018). Validation of ANAM for cognitive screening in a mixed clinical sample. Appl. Neuropsychol. Adult 25, 366–375. 28448160 10.1080/23279095.2017.1314967

[28] Teasdale, G.M., Pettigrew, L.E., Wilson, J.T., Murray, G., and Jennett, B. (1998). Analyzing outcome of treatment of severe head injury: a review and update on advancing the use of the Glasgow Outcome Scale. J. Neurotrauma 15, 587–597. 9726258 10.1089/neu.1998.15.587

[29] Diener, E., Emmons, R.A., Larsen, R.J., and Griffin, S. (1985). The Satisfaction With Life Scale. J. Pers. Assess. 49, 71–75. 16367493 10.1207/s15327752jpa4901_13

[30] Buysse, D.J., Reynolds, C.F. III, Monk, T.H., Berman, S.R., and Kupfer, D.J. (1989). The Pittsburgh Sleep Quality Index: a new instrument for psychiatric practice and research. Psychiatry Res. 28, 193–213. 2748771 10.1016/0165-1781(89)90047-4

[31] Fichtenberg, N.L., Zafonte, R.D., Putnam, S., Mann, N.R., and Millard, A.E. (2002). Insomnia in a post-acute brain injury sample. Brain Inj. 16, 197–206. 11874613 10.1080/02699050110103940

[32] World Health Organization. (2010). International Classification of Disease, 10th Review. World Health Organization: Geneva, Switzerland.

[33] Killgore, W.D. (2010). Effects of sleep deprivation on cognition. Prog. Brain Res. 185, 105–129. 21075236 10.1016/B978-0-444-53702-7.00007-5

[34] Mao, T., Dinges, D., Deng, Y., Zhao, K., Yang, Z., Lei, H., Fang, Z., Yang, F.N., Galli, O., Goel, N., Basner, M., and Rao, H. (2021). Impaired vigilant attention partly accounts for inhibition control deficits after total sleep deprivation and partial sleep restriction. Nat. Sci. Sleep 13, 1545–1560. 34557048 10.2147/NSS.S314769 PMC8455079

[35] Rasch, B., and Born, J. (2013). About sleep's role in memory. Physiol. Rev. 93, 681–766. 23589831 10.1152/physrev.00032.2012 PMC3768102

[36] Plihal, W., and Born, J. (1997). Effects of early and late nocturnal sleep on declarative and procedural memory. J. Cogn. Neurosci. 9, 534–547. 23968216 10.1162/jocn.1997.9.4.534

[37] Wagner, U., Hallschmid, M., Verleger, R., and Born, J. (2003). Signs of REM sleep dependent enhancement of implicit face memory: a repetition priming study. Biol. Psychol. 62, 197–210. 12633978 10.1016/s0301-0511(02)00125-4

[38] Gais, S., and Born, J. (2004). Declarative memory consolidation: mechanisms acting during human sleep. Learn. Mem. 11, 679–685. 15576885 10.1101/lm.80504 PMC534696

[39] Ribeiro, S., Shi, X., Engelhard, M., Zhou, Y., Zhang, H., Gervasoni, D., Lin, S.C., Wada, K., Lemos, N.A., and Nicolelis, M.A. (2007). Novel experience induces persistent sleep-dependent plasticity in the cortex but not in the hippocampus. Front. Neurosci. 1, 43–55. 18982118 10.3389/neuro.01.1.1.003.2007 PMC2577304

[40] Parcell, D.L., Ponsford, J.L., Redman, J.R., and Rajaratnam, S.M. (2008). Poor sleep quality and changes in objectively recorded sleep after traumatic brain injury: a preliminary study. Arch. Phys. Med. Rehabil. 89, 843–850. 18452730 10.1016/j.apmr.2007.09.057

[41] Schreiber, S., Barkai, G., Gur-Hartman, T., Peles, E., Tov, N., Dolberg, O.T., and Pick, C.G. (2008). Long-lasting sleep patterns of adult patients with minor traumatic brain injury (mTBI) and non-mTBI subjects. Sleep Med. 9, 481–487. 17638592 10.1016/j.sleep.2007.04.014

[42] Mantua, J., Mahan, K.M., Henry, O.S., and Spencer, R.M. (2015). Altered sleep composition after traumatic brain injury does not affect declarative sleep-dependent memory consolidation. Front. Hum. Neurosci. 9, 328. 26097451 10.3389/fnhum.2015.00328 PMC4456580

[43] Ribeiro, S. (2012). Sleep and plasticity. Pflugers Arch. 463, 111–120. 21947578 10.1007/s00424-011-1031-5 PMC3256318

[44] Gorgoni, M., D'Atri, A., Lauri, G., Rossini, P.M., Ferlazzo, F., and De Gennaro, L. (2013). Is sleep essential for neural plasticity in humans, and how does it affect motor and cognitive recovery? Neural Plast. 2013, 103949. 23840970 10.1155/2013/103949 PMC3693176

[45] Zunzunegui, C., Gao, B., Cam, E., Hodor, A., and Bassetti, C.L. (2011). Sleep disturbance impairs stroke recovery in the rat. Sleep 34, 1261–1269. 21886364 10.5665/SLEEP.1252 PMC3157668

[46] Facchin, L., Schone, C., Mensen, A., Bandarabadi, M., Pilotto, F., Saxena, S., Libourel, P.A., Bassetti, C.L.A., and Adamantidis, A.R. (2020). Slow waves promote sleep-dependent plasticity and functional recovery after stroke. J. Neurosci. 40, 8637–8651. 33087472 10.1523/JNEUROSCI.0373-20.2020 PMC7643301

[47] Fultz, N.E., Bonmassar, G., Setsompop, K., Stickgold, R.A., Rosen, B.R., Polimeni, J.R., and Lewis, L.D. (2019). Coupled electrophysiological, hemodynamic, and cerebrospinal fluid oscillations in human sleep. Science 366, 628–631. 31672896 10.1126/science.aax5440 PMC7309589

[48] Hauglund, N.L., Kusk, P., Kornum, B.R., and Nedergaard, M. (2020). Meningeal lymphangiogenesis and enhanced glymphatic activity in mice with chronically implanted EEG electrodes. J. Neurosci. 40, 2371–2380. 32047056 10.1523/JNEUROSCI.2223-19.2020 PMC7083292

[49] Iliff, J.J., Wang, M., Liao, Y., Plogg, B.A., Peng, W., Gundersen, G.A., Benveniste, H., Vates, G.E., Deane, R., Goldman, S.A., Nagelhus, E.A., and Nedergaard, M. (2012). A paravascular pathway facilitates CSF flow through the brain parenchyma and the clearance of interstitial solutes, including amyloid beta. Sci. Transl. Med. 4, 147ra111. 10.1126/scitranslmed.3003748 PMC3551275 22896675

[50] Louveau, A., Plog, B.A., Antila, S., Alitalo, K., Nedergaard, M., and Kipnis, J. (2017). Understanding the functions and relationships of the glymphatic system and meningeal lymphatics. J. Clin. Invest. 127, 3210–3219. 28862640 10.1172/JCI90603 PMC5669566

[51] Harrison, I.F., Ismail, O., Machhada, A., Colgan, N., Ohene, Y., Nahavandi, P., Ahmed, Z., Fisher, A., Meftah, S., Murray, T.K., Ottersen, O.P., Nagelhus, E.A., O'Neill, M.J., Wells, J.A., and Lythgoe, M.F. (2020). Impaired glymphatic function and clearance of tau in an Alzheimer's disease model. Brain 143, 2576–2593. 32705145 10.1093/brain/awaa179 PMC7447521

[52] Iliff, J.J., Chen, M.J., Plog, B.A., Zeppenfeld, D.M., Soltero, M., Yang, L., Singh, I., Deane, R., and Nedergaard, M. (2014). Impairment of glymphatic pathway function promotes tau pathology after traumatic brain injury. J. Neurosci. 34, 16180–16193. 25471560 10.1523/JNEUROSCI.3020-14.2014 PMC4252540

[53] Bolte, A.C., Dutta, A.B., Hurt, M.E., Smirnov, I., Kovacs, M.A., McKee, C.A., Ennerfelt, H.E., Shapiro, D., Nguyen, B.H., Frost, E.L., Lammert, C.R., Kipnis, J., and Lukens, J.R. (2020). Meningeal lymphatic dysfunction exacerbates traumatic brain injury pathogenesis. Nat. Commun. 11, 4524. 32913280 10.1038/s41467-020-18113-4 PMC7483525

[54] Ren, Z., Iliff, J.J., Yang, L., Yang, J., Chen, X., Chen, M.J., Giese, R.N., Wang, B., Shi, X., and Nedergaard, M. (2013). ‘Hit & Run’ model of closed-skull traumatic brain injury (TBI) reveals complex patterns of post-traumatic AQP4 dysregulation. J. Cereb. Blood Flow Metab. 33, 834–845. 23443171 10.1038/jcbfm.2013.30 PMC3677112

[55] Sullan, M.J., Asken, B.M., Jaffee, M.S., DeKosky, S.T., and Bauer, R.M. (2018). Glymphatic system disruption as a mediator of brain trauma and chronic traumatic encephalopathy. Neurosci. Biobehav. Rev. 84, 316–324. 28859995 10.1016/j.neubiorev.2017.08.016

[56] Shokri-Kojori, E., Wang, G.J., Wiers, C.E., Demiral, S.B., Guo, M., Kim, S.W., Lindgren, E., Ramirez, V., Zehra, A., Freeman, C., Miller, G., Manza, P., Srivastava, T., De Santi, S., Tomasi, D., Benveniste, H., and Volkow, N.D. (2018). beta-Amyloid accumulation in the human brain after one night of sleep deprivation. Proc. Natl. Acad. Sci. U. S. A. 115, 4483–4488. 29632177 10.1073/pnas.1721694115 PMC5924922

[57] Kang, J.E., Lim, M.M., Bateman, R.J., Lee, J.J., Smyth, L.P., Cirrito, J.R., Fujiki, N., Nishino, S., and Holtzman, D.M. (2009). Amyloid-beta dynamics are regulated by orexin and the sleep-wake cycle. Science 326, 1005–1007. 19779148 10.1126/science.1180962 PMC2789838

[58] Piantino, J., Schwartz, D.L., Luther, M., Newgard, C., Silbert, L., Raskind, M., Pagulayan, K., Kleinhans, N., Iliff, J., and Peskind, E. (2021). Link between mild traumatic brain injury, poor sleep, and magnetic resonance imaging: visible perivascular spaces in veterans. J. Neurotrauma 38, 2391–2399. 33599176 10.1089/neu.2020.7447 PMC8390772

